# Notes on Pepsine—A Much Abused and Misrepresented Remedy

**Published:** 1880-09

**Authors:** Carl Jungk


### NOTES ON PEPSINE—A MUCH ABUSED AND MISREPRESENTED REMEDY.

BY CARL JUNGK, Ph. D.

As there are individuals, who, on the appearance of a newly introduced remedy, at once discover wonderful curative properties in it, and enthusiastically laud it to the skies, so there are minds who live in an atmosphere of chronic doubt and distrust, and from principle are continually engaged in a warfare against all new agents introduced to therapeutics through pharmaceutic mediation. Both classes may at times be right, yet just as often be in the wrong, and it would seem very desirable to observe the golden mean between the two extremes. We need only, for an instance, refer to the career of salicylic acid. What astonishing and wonderful properties were at first attributed to this article, and with what enthusiasm was it received? Technologists and other scientific men were completely carried away with it, and related wonderful accounts of its antiseptic virtues. It was consequently very largely used in nearly all branches of industry, where it could find application. But how have these expectations dwindled to a reality of insignificant proportions, and how many have not been disappointed who placed too much reliance on its potency and virtues? Whether the reason for these disappointments is to be looked for in the frequently incorrect and faulty methods of its application, or in the properties of the body itself, which may have been overestimated as an antiseptic, is a question which may be dismissed for the present; but the one fact remains, that medical science has at least received the benefit of an addition to its armamentarium in a remedy for acute articular rheumatism, possessing considerable value, though not to be regarded as a specific.

From a speculative standpoint, might it not be possible that the artificially-produced salicylic acid is only isomeric with the natural; that it is to be regarded rather as carbonic acid whose oxygen is partly substituted by phenol, and whose contact with ferments provokes a decomposition back to its original molecular factors, *i. e.* to carbonic acid and carbolic acid; and that where the causes of this retrograde decomposition are absent, no antiseptic action is exhibited. According to such a view we should look to the liberated carbolic acid as the cause of its activity.

It is far from my purpose to set up such a theory in antagonism to the views at present received, but certain observations have led me to consider the possibility of such a relation, withcut as yet having endeavored to demonstrate it experimentally.

The object of the above introductory statements is to call attention to the inappropriateness and injustice of the alternate over and under estimate of such new remedies, and for further instance I need only refer to chloral hydrate, chloroform, benzoate of soda, and of recent date and interests, to bromide of ethyl. The latter, after a very successful period of use in France and Germany, a period remarkable for its immunity from danger, is all at once menaced, soon after its introduction here, by a risk of being discarded. At present it seems as if still another remedy, pepsine, were to be subjected to villification and contumely and perhaps eventually, though undeservedly, meet the fate awarded to pretenders. Many of our contemporaries, both at home and abroad, have declared a war of extermination against the innocent remedy, while at the same time others have been as prompt to take up arms in its defense.

The results obtained by a number of skilled scientific experimenters, by men the mention of whose names alone would inspire confidence in the results ef their labors, are to count as nothing. The very fact that pepsine in acidulated solution is capable of transforming albumen into peptone is to be disputed. That five grains of saccharated pepsine of normal strength are only able to dissolve two grains of albumen instead of sixty grains, as has heretofore been believed, is to be accepted as individual error of observation in the latter case, and the conclusion adopted that all results in harmony with this standard are incorrect, and only the last observer infallible. We can well exclaim with Gœthe's Faust, "True, I hear the tidings, though credence fails me." It is wonderful what astonishingly incorrect observers such men as Schwan, Pappenheim, Lehmann, Bidder and Schmidt, Scheffer, etc., must have been if these latter assertions are true! Assertions are easily made which, with a stroke of the pen, are to annihilate theories based on experience and physiological demonstration; but substantial pooof of logical correctness is quite another thing.

It would be well, at this point, to review, in short, the history of pepsine, and next to devote a cursory inspection to the article itself. The presence of a peculiar gastric ferment or digestive principle was first suspected by Eberle, and Schwan succeeded in isolating a substance from the gastric juice by digestion with water, and producing a precipitation with mercuric chloride, and which, on removal of the mercury, a principle which exercised a solvent or digestive action on coagulated protein substances. He named this substance pepsine (after the Greek *pepsis*, digestion or carbonization.)

Wasman used the mucous membranes of hogs' stomachs, and digested them repeatedly with water at 30° to 35° C., until putrefaction commenced. The mixed, clear liquors were precipitated with lead or mercury salts, and the metals removed as sulphides, the resulting filtrate concentrated at a low temperature, and the liquid then precipitated with alcohol. He states that one part of such pepsine in weak acidulated solution was capable of dissolving 60,000 parts of coagulated egg-albumen in six to eight hours. This method of preparation has since been adhered to in Boudault's pepsine.

Pappenheim, who also devoted considerable attention to the experimental preparation of pepsine, states that it is precipitable by the sulphates of iron and copper, but soluble is excess of the reagents. His pepsine was not free from albumen.

Bidder and Schmidt treated the gastric juice with lime, evaporated the filtrate to a syrupy consistence, precipitated with alcohol, and obtained a tolerably pure article. Their preparation gave, with an excess only of corrosive sublimate, a permanent precipitate, and could be heated to 107° C., without decomposition.

A few elementary analyses of pepsine have been made, but at present these possess but little value. Only when it shall have become possible to isolate the gastric ferment in a state of purity, will such an analysis be of service. Schmidt's assertion that pepsine combines with chlorohydric acid, forming a compound acid named by him chloropepsinhydric acid, and that it acts as such, has so far not been negatived, and seems to have some foundation for its support. The processes above mentioned with others may furnish a digestive remedy which is very good when fresh, but which deteriorates, more or less, in the course of time, losing its activity.

Too much precipitation, solution, re-precipitation, evaporation and other like treatment, cannot improve the quality of pepsine very much, as it must be regarded in the light of a ferment, and possessing the properties of a ferment. Such treatment applied to yeast would certainly deprive it of the greater part, if not of all, of its distinctive character and properties.

Professor Scheffer, of Louisville, was the first who practically realized the views just mentioned, by elaborating a simple and economical process for the preparation of pepsine. His principal endeavor was to accomplish the contact of the peptic principle with an acidulated liquid until solution and clarification had been effected and then separate it from its solution in a simple manner, and at the same time by an agent having preservative properties. The majority of manufacturers now prepare it, in the main, according to Scheffer's process, but the methods of drying or concentrating the pepsin may differ, and thus, perhaps affect in some degree the quality of the resulting preparation. An article skillfully prepared, with all necessary precautions, will usually dissolve a larger quantity of coagulated albumen than has been adopted as the standard, but the influence of age has a tendency to diminish its power. For natural reasons a very concentrated pepsin will lose its activity more rapidly than a saccharated preparation, as in the latter the preservative properties of milk-sugar exert an influence in delaying change or decomposition. The idea of furnishing very concentrated pepsine to the trade is therefore impractical.

If the pepsine is first mixed with milk-sugar while it is still moist the drying proceeds with greater rapidity, which rapidity is in direct proportion to the quantity of milk-sugar added, the addition added also increasing the permanency of the preparation. It is of little consequence whether one, ten or twenty grains of a given article of pepsine be administered, but certainly it is better to give twenty grains of an active though mild preparation, than one grain of an article professing to be concentrated, but at the same time of doubtful activity. Pepsine should, whenever possible, be administered in combination with acids, of which lactic acid and muriatic are the best. If we mix fifty grains of a carefully prepared fresh saccharated pepsine with sixty minims of lacic acid, and sixty minims of mutric acid, and add four ounces of chopped beef, free from fat, and one and one-half pints of water, then allowing the mixture to stand for three hours, with frequent agitations at a temperature of 110 to 115o F., the meat will pass into solution, with a residue of but about two drachms, equaling six and one-fourth per cent. If to one part acidulated pepsine, we add from 50,000 to 80,000 parts of fresh milk, in a short time the entire quantity will coagulate; if the process is repeated without pepsine, with the same quantity of acid, the milk will preserve its fluidity.

The efficacy of pepsine is dyspepsia, colic, stomachic cramps, and in general in disturbances where the digestive organs are concerned, has been too generally attested by competent observers to leave any room for doubt. In cases of over-indulgence in food or wine, it is an unexcelled remedy, producing relief frequently in five or ten minutes; it has no superior as a dietic remedy, while it has achieved a reputation, according to statements from good authorities, in the vomiting of pregnancy.

The best method for preserving the activity of pepsine for long periods is to present it in acidulated solution with glycerine; such a preparation should in all cases have the preference, although it may in course of time acquire more or less color.

The most concentrated permanent form of pepsine, in which it is possible to present it, has about the following composition:

| | | |
|---|---|---|
| Water . . . . . . . . . . | 5 | parts. |
| Ash (soluble in water) . . . | 15 | " |
| Ash (insoluble in water) . . . | 2 | " |
| Sugar-milk . . . . . . . | 62 | " |
| Pepsine (organic matter) . . | 16 | " |
| | 100 | |

Four parts of such a preparation, mixed with one part of milk-sugar, gave an article which, shortly after its manufacture, was capable of dissolving for each part employed, 100 parts coagulated albumen, previously chopped, the process being even completed, when continual agitation was employed, in one-half hour. After the lapse of three months, it had lost, in spite of very careful preservation. a part of its solvent properties; when, however, it was digested for twenty-four hours with some muriatic acid, lactic acid, and glycerine, it was again capable of dissolving coagulated albumen in the same proportion as before, Muriatic acid itself possesses a slight solvent property on albumen, but is not capable of effecting its transformation into peptone. It is far from my purpose to convey the impression that there are no worthless preparations in the market; such are almost invariably made pleasing to the eye without regard to their therapeutic value. The existence of worthless preparations is, however, no valid reason for denying entirely the digestive activity of pepsine on albumen and analagous substances, and its power of transforming such into peptone.—*Therapeutic Gazette.*

